# Compatible Stability and Aerosol Characteristics of Atrovent^®^ (Ipratropium Bromide) Mixed with Salbutamol Sulfate, Terbutaline Sulfate, Budesonide, and Acetylcysteine

**DOI:** 10.3390/pharmaceutics12080776

**Published:** 2020-08-15

**Authors:** Yiting Chen, Shilin Du, Zhirui Zhang, Wenxiu He, Enhao Lu, Rui Wang, Xianyi Sha, Yan Ma

**Affiliations:** 1 Key Laboratory of Smart Drug Delivery, Ministry of Education, School of Pharmacy, Fudan University, Shanghai 201203, China; 18211030015@fudan.edu.cn (Y.C.); 16301030052@fudan.edu.cn (Z.Z.); 18111030032@fudan.edu.cn (W.H.); 19211030026@fudan.edu.cn (E.L.); 19111030038@fudan.edu.cn (R.W.); 2 Department of Emergency Medicine, Zhongshan Hospital, Fudan University, Shanghai 200032, China; du.shilin@zs-hospital.sh.cn; 3 The Institutes of Integrative Medicine of Fudan University, Shanghai 200040, China; 4 Shanghai Mental Health Center, School of Medicine, Shanghai Jiaotong University, Shanghai 200030, China

**Keywords:** Atrovent^®^, Ventolin^®^, Bricanyl^®^, Pulmicort^®^, Fluimucil^®^, ipratropium, nebulization, compatibility, aerosol characteristics, simultaneous inhalation

## Abstract

(1) Background: It is common practice in the treatment of respiratory diseases to mix different inhalation solutions for simultaneous inhalation. At present, a small number of studies have been published that evaluate the physicochemical compatibility and aerosol characteristics of different inhalation medications. However, none of them studied Atrovent^®^. Our work aims to address the lack of studies on Atrovent^®^. (2) Methods: Portions of admixtures were withdrawn at certain time intervals after mixing and were tested by pH determination, osmolarity measurement, and high-performance liquid chromatography (HPLC) assay of each active ingredient as measures of physicochemical compatibility. The geometrical and aerosol particle size distribution, active drug delivery rate, and total active drug delivered were measured to characterize aerosol behaviors. (3) Results: During the testing time, no significant variation was found in the pH value, the osmotic pressure, or the active components of admixtures. With the increase in nebulization volume after mixing, fine particle dose (FPD) and total active drug delivered showed statistically significant improvements, while the active drug delivery rate decreased compared to the single-drug preparations. (4) Conclusions: These results endorse the physicochemical compatibility of Atrovent^®^ over 1 h when mixed with other inhalation medications. Considering aerosol characteristics, simultaneous inhalation is more efficient.

## 1. Introduction

Aerosol therapy is defined as an inhalation treatment that utilizes inhaler devices to transform pharmaceutical agents into aerosol form [1]. The specific features of the pulmonary route, such as large absorption area, thin alveolar-capillary wall, low enzyme activity, and avoidance of the first-pass effect make pulmonary drug delivery advantageous in terms of fast absorption and high bioavailability. Thus, aerosol therapy is widely used in the diagnosis and treatment of lung pathology, including, but not limited to, chronic obstructive pulmonary disease (COPD), asthma, and cystic fibrosis (CF) [2]. Furthermore, from the perspective of clinical medicine, aerosol therapy is quite suitable for children, aged patients, and patients with difficulties in swallowing. Frequently used inhaler devices are classified into three types: pressurized metered dose inhalers (pMDI), dry powder inhalers (DPI), and nebulizers. Among these, the use of nebulizers is well-established, seen extensively both in hospitals and domiciliary settings [1]. Several classes of respiratory therapeutic medications, including short-acting beta_2_ receptor agonists (SABA, e.g., salbutamol sulfate), long-acting beta_2_ receptor agonists (LABA, e.g., formoterol fumarate), short-acting muscarinic antagonists (SAMA, e.g., ipratropium bromide), long-acting muscarinic antagonists (LAMA, e.g., tiotropium bromide), and inhaled corticosteroids (ICS, e.g., budesonide), have been formulated into inhalation solutions as mainstream medicines for COPD and asthma [3].

The pathogenesis of COPD and asthma is so complicated that the target sites are multiple and combination therapies are often necessary. According to the suggestions of the Global Initiative for Chronic Obstructive Lung Disease (GOLD) and the Global Initiative for Asthma (GINA), dual combination therapy of LAMA/LABA or ICS/LABA is optimal for patients who are suffering from poorly controlled symptoms or frequent exacerbation. If the symptoms persist or the patients are at a high risk of exacerbation after undergoing dual combination therapy, treatment should be escalated to trimodal therapy of ICS/LABA/LAMA [4,5].

It is worth noting that patients prescribed concomitant nebulized medications prefer admixing multiple drugs for simultaneous inhalation in order to reduce administration time and increase patients’ compliance. Although most of the previous studies have proven the advantages of combination therapies in terms of addictive/synergistic pharmacological activities [6,7,8,9], the potential incompatibility between excipients and/or drugs in different commercial products may lead to adverse effects and toxicity, which should not be ignored. Nevertheless, little research has been carried out on the physicochemical compatibility of mixtures [10,11], especially on aerosol characteristics [12,13,14], which may significantly impact drug absorption and subsequently inhalation efficacy. A study has shown that Pulmozymel^®^ (brand of dornase alfa) should not be used along with Atrovent^®^ (brand of ipratropium) or Sultanol^®^ (brand of albuterol) because benzalkonium chloride (BAC) and disodium edetate used as excipients in Atrovent^®^ or Sultanol^®^ may destroy the integrity of dornase alfa, thereby causing microbiological instability [15]. Likewise, cloudiness that occurs when mixing colistin or cromolyn with Atrovent^®^ or Sultanol^®^ is attributed to BAC [10]. It is necessary to monitor the changes in pH and osmolality while mixing, which influence the tolerability of inhalation solutions. The extreme pH value (<2.6 or >10.0) will cause tissue irritation. The best osmolality is between 150 and 1200 mOsm/kg to prevent coughing or bronchoconstriction [16,17]. Another crucial factor that affects drug delivery efficiency to the deep alveolar region of the lung is the aerosol particle size distribution, which is often measured with metrics such as fine particle dose (FPD), fine particle fraction (FPF), and mass medium aerodynamic diameter (MMAD). To obtain optimal aerosol delivery, the aerodynamic diameter should be 1–5 µm, for the reason that particles smaller than 0.5 µm are more likely to be exhaled via the Brownian movement and larger particles (>5 µm) easily deposit in the upper respiratory tract [18,19,20,21]. The aerosol characteristics will be affected by the physical characteristics and compositions of formulations [22]. Additionally, solutions and suspensions for nebulization behave differently with regard to heterogeneous distribution [23]. Taken together, these findings suggest that simultaneous inhalations of different solutions/suspensions may influence the deposition of inhaled aerosols into the lung by altering aerosol particle size distribution and simultaneous inhalation [24,25].

Atrovent^®^ (ipratropium bromide), the most commonly used SAMA, is a bronchodilator that works via blockade of muscarinic cholinergic receptors and is clinically used in combination with other inhalation solutions/suspensions for enhanced treatment efficacy [26]. For example, concomitant therapy containing ipratropium bromide with albuterol or salbutamol can be taken as relief medication or used for maintenance therapy [27]. It was reported that ipratropium bromide combined with salmeterol exhibited excellent improvements in post-bronchodilator forced expiratory volume in 1 s (FEV_1_) [28]. However, until now, there has been no systematic study on the physicochemical compatibility and aerosol characteristics of Atrovent^®^ mixed with other inhalation solutions/suspensions. Therefore, in this work, we attempt to fill this research gap in order to create a useful reference for medical personnel.

## 2. Materials and Methods

### 2.1. Materials

Atrovent^®^ (ipratropium bromide solution for inhalation, 250 µg/2 mL per vial; Boehringer Ingelheim, Ingelheim am Rhein, Germany), Ventolin^®^ (salbutamol sulfate inhalation solution, 5 mg/2.5 mL per vial; GlaxoSmithKline, Boronia Vic 3155, Australia), Bricanyl^®^ (terbutaline sulfate solution for nebulization, 5 mg/2 mL; AstraZeneca, Cambridge, UK), Pulmicort^®^ (budesonide suspension for inhalation, 1 mg/2 mL per vial; AstraZeneca, Cambridge, UK), and Fluimucil^®^ (*N*-acetylcysteine(NAC) solution for inhalation, 0.3 g/3 mL per ampule; Zambon, Bresso, Italy) were available commercially. Reference substances including ipratropium bromide, salbutamol sulfate, and terbutaline sulfate were purchased from Dalian Meilun Biotechnology Co., Ltd. (Dalian, China), while reference substances of budesonide and acetylcysteine were obtained from National Institutes for Food and Drug Control (Beijing, China). Sodium dihydrogen phosphate was purchased from Beijing Solarbio Science & Technology Co., Ltd. (Beijing, China). Phosphoric acid was purchased from Sinopharm Chemical Reagent Co., Ltd. (Shanghai, China). Acetonitrile (HPLC grade) was purchased from Merck KGaA (Darmstadt, Germany). Deionized water was produced by a Milli-Q water purification system (Millipore, Bedford, MA, USA).

### 2.2. Preparation of Samples

The binary admixtures were prepared by mixing a single dose of Atrovent^®^ with a single dose of Ventolin^®^, Bricanyl^®^, Pulmicort^®^, and Fluimucil^®^ separately, while the ternary mixture contained one dose of Atrovent^®^, Ventolin^®^, and Pulmicort^®^. All the admixtures were oscillated gently to ensure uniformity and homogeneity. The preparation and testing processes were conducted at ambient room temperature to simulate real-world usage. For physical compatibility assessments, a single dose of each drug served as the control group. For aerosol characteristics, two control groups were set: a single dose of each drug and each drug diluted with saline solution (0.9% NaCl). Unless otherwise stated, all admixtures were prepared in quadruplicate and all the experiments following were repeated five times.

The nebulization of all samples was conducted by a PARI Turboboy N compressor/LC Plus nebulizer (PARI GmbH, Starnberg, Germany). To determine the nebulization time before formal experiments, the tested sample was placed in a dried nebulization cup connected with the PARI Turboboy N compressor/LC Plus nebulizer and continuously nebulized until no more aerosol formed. The whole process was timed with a stopwatch and recorded (any fraction of one minute was counted as one minute to ensure complete nebulization). The volume and nebulization times of all samples are summarized in Table 1.

### 2.3. Physical Compatibility Assessments

Portions of samples were withdrawn from each mixture at predetermined time intervals (0(T_0_), 0.25(T_0.25_), 0.5(T_0.5_), and 1(T_1_) hour after preparation). Then, the pH value was measured using a calibrated pH meter (FiveEasy Plus™ FE28-Meter, Mettler Toledo, Greifensee, Switzerland) and the osmolality was analyzed by the freezing point depression method (Osmomat 030, Gonotec, Berlin, Germany).

### 2.4. Chemical Stability Assessments

To evaluate chemical stability, 100 µL samples collected from each solution at 0(T_0_), 0.25(T_0.25_), 0.5(T_0.5_), and 1(T_1_) hour after mixing were diluted to certain proportions with mobile phase and were immediately quantitatively analyzed on an Agilent 1260 HPLC system equipped with a quaternary pump, a vacuum degasser, an autosampler, and a diode array detector (DAD) to calculate the concentration of each active component. For all analyses, the HPLC column used a DiKMA Spursil C18 column (5 μm, 250 × 4.6 mm) with a flow rate of 1 mL/min and a column temperature of 35 °C. Mobile phase A was sodium dihydrogen phosphate (NaH_2_PO_4_) buffer (3.17 g NaH_2_PO_4_ and 0.23 g phosphoric acid were dissolved in 800 mL deionized water; then, the pH value of solutions was adjusted to 3.2 and deionized water was used to adjust the final volume to 1 L.). Acetonitrile was served as mobile phase B. The ratio of the mobile phase, injection volume, run time, and the detection wavelength are listed in Table 2. The concentration of each active component was calculated by building standard curves.

### 2.5. Particle Size Distribution Characterization by Laser Diffraction

The geometrical particle size of a single dose of each drug or the prepared mixtures was characterized by laser diffraction using the Sympatec HELOS/BF laser diffraction meter (Sympatec GmbH, Clausthal-Zellerfeld, Germany). Before the first experiment, the background was tested in the dark condition for calibration. For testing, the nebulization cup, containing a single dose of each drug or the prepared mixtures, was positioned so that the laser beam went through the center of the spray area and so as to keep a vertical distance between the nozzle of nebulization cup and the center of laser light source of 6 cm. The instrument parameters and detailed settings were as follows: lens: R1 (0.1/0.18–0.35μm); start: 10 s after c.opt ≥ 3.0%; stop: 30 s after start or 0.0 s after c.opt ≥ 3.0%; time base: 5 ms. A series of experimental data, X10 (particle size which corresponds to 10% of cumulative volume distribution), X50 (particle size which corresponds to 50% of cumulative volume distribution), X90 (particle size which corresponds to 90% of cumulative volume distribution), volume median droplet diameter (VMD), was calculated by the data-processing software WINDOX (Sympatec, Clausthal-Zellerfeld, Germany).

### 2.6. Aerosol Particle Distribution Measurement by the Next-Generation Impactor

The next-generation impactor (NGI, Copley Scientific, Nottingham, UK) with seven stages with progressively decreasing cut-off diameters and a Micro-Orifice Collector (MOC, Copley Scientific, Nottingham, UK) were used to determine the aerosol particle distribution. Before the inception of the experiment, a leak test was carried out and the flow rate was set to 15 L/min within ±5% by a flow meter (flow meter model DFM4, Copley Scientific, Nottingham, UK). All the NGI experiments were performed in an NGI cooler (Copley Scientific, Nottingham, UK) at a controlled temperature of 5 ± 1.5 °C, with a pre-cooling time of at least 90 min. Following the manufacturers’ instructions, the NGI was attached with the induction port and connected with a vacuum pump (high capacity pump model HCP5, Copley Scientific, Nottingham, UK). The admixtures were prepared separately in the nebulization cup, connected with the induction port by a suitable mouthpiece adapter. After corresponding aerosol time, the nebulizer (PARI Turboboy N compressor/LC Plus nebulizer, PARI GmbH, Starnberg, Germany) and the pump were switched off in turn. The samples in the induction port and removable impaction cups were collected into suitable volumetric flasks with deionized water/acetonitrile (3:1) and were analyzed with HPLC. The results of the various tests, including FPD, FPF, and MMAD, were calculated by Copley inhaler testing data analysis software (CITDAS, Copley Scientific, Nottingham, UK).

### 2.7. Active Drug Delivery Rate and Total Active Drug Delivered

The active drug delivery rate and total active drug delivered were tested by a delivered dose sampling apparatus consisting of a breathing simulator (breathing simulator model BRS 1100, Copley Scientific, Nottingham, UK), an angle adapter, an inhalation filter, and a filter holder. The nebulization cup containing a single dose of drug or admixtures prepared as above was inserted into a mouthpiece adapter to connect with the delivered dose sampling apparatus and was positioned upright. The breathing profile that is representative of an adult was set as follows: tidal volume was 500 mL; frequency was 15 cycles/min; waveform was sinusoidal; inhalation/exhalation (I/E) ratio was 1:1. Following the completion of the assembly and the set, the nebulizer and the breathing simulator were switched on at the same time and also were paused simultaneously after 60 s. The drug deposited on the filter and filter holder was collected with deionized water/acetonitrile (3:1); then, a new fresh filter was placed, and the test was maintained until the preset nebulization time ran out. In the same way, the drug was collected and analyzed with the HPLC method. Finally, we divided the drug content collected from the first filter and filter device by 1 min to obtain the active drug delivery rate. The total active drug delivered was defined as the sum of the drug content in all filters.

## 3. Results

### 3.1. Physicochemical Compatibility

**Figure 1:**
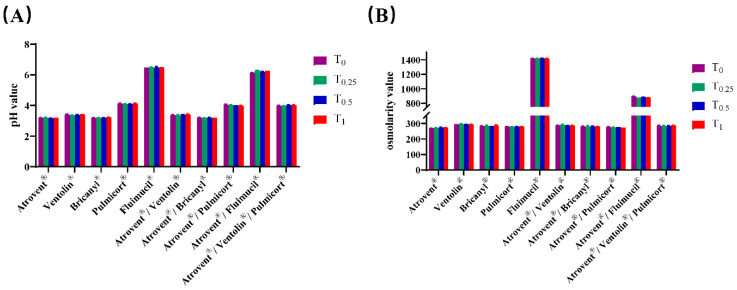
The pH value (**A**) and osmolarity value (**B**) of test samples over time. Experiments were replicated 5 times.

The changes in pH value and osmolarity value of test samples over time are presented in Figure 1. The pH and osmolarity of admixtures seem to be a compromise value between the value of each component. Furthermore, for all admixtures, these two values showed a small variation that can be ignored within the 1 h testing time.

**Figure 2:**
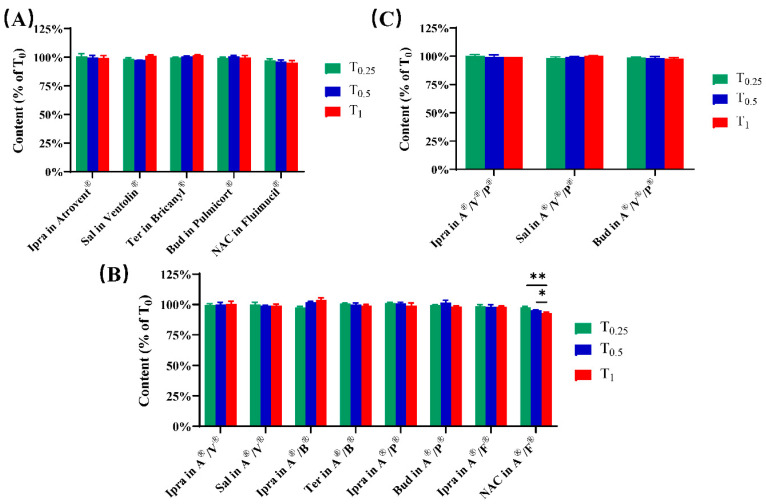
The content of active components in single dose of each drug (**A**), binary admixtures (**B**), and ternary admixtures (**C**) over time (mean ± s.d. shown). *n* = 5 in each group. * *p* < 0.05 with analysis of variance (ANOVA), ** *p* < 0.01 with ANOVA. Abbreviations: Ipra—ipratropium bromide; Sal—salbutamol sulfate; Ter—terbutaline sulfate; Bud—budesonide; NAC—N-acetylcysteine; A^®^/V^®^—Atrovent^®^/Ventolin^®^; A^®^/B^®^—Atrovent^®^/Bricanyl^®^; A^®/^P^®^—Atrovent^®^/Pulmicort^®^; A^®^/F^®^—Atrovent^®^/ Fluimucil^®^; A^®^/V^®^/P^®^—Atrovent^®^/Ventolin^®^/Pulmicort^®^.

**Figure 3:**
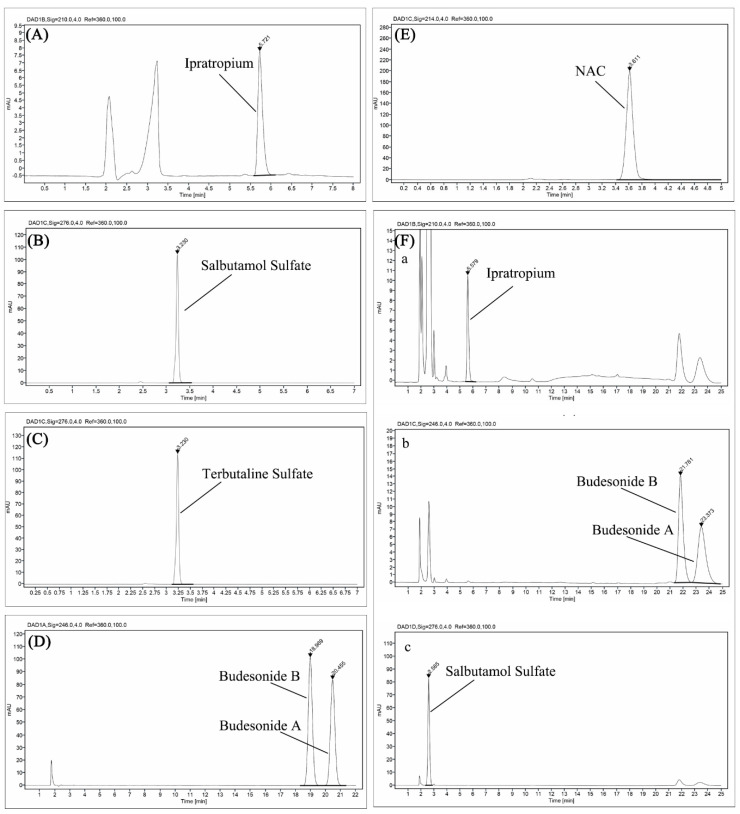
HPLC chromatograms of (**A**) Atrovent^®^ (210 nm), (**B**) Ventolin^®^ (276 nm), (**C**) Bricanyl^®^ (276 nm), (**D**) Pulmicort^®^ (246 nm), (**E**) Fluimucil^®^ (214 nm), and (**F**) Atrovent^®^/Ventolin^®^/Pulmicort^®^ ((**a**) 210 nm for ipratropium bromide, (**b**) 246 nm for budesonide, and (**c**) 276 nm for salbutamol sulfate)).

**Figure 4:**
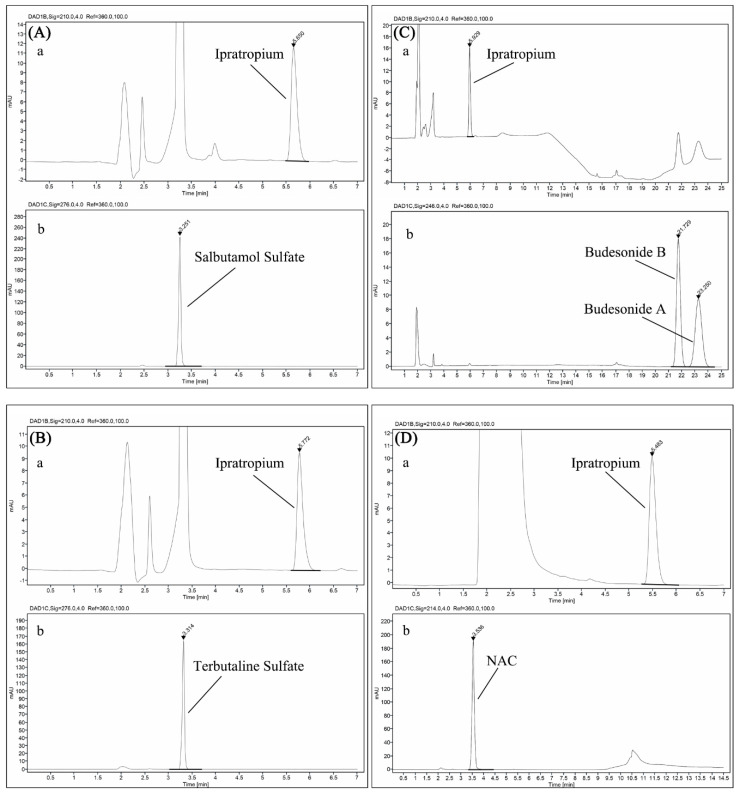
HPLC chromatograms of (**A**) Atrovent^®^/Ventolin^®^ ((**a**) 210 nm for ipratropium bromide, (**b**) 276 nm for salbutamol sulfate)), (**B**) Atrovent^®^/ Bricanyl^®^ ((**a**) 210 nm for ipratropium bromide, (**b**) 276 nm for terbutaline sulfate)), (**C**) Atrovent^®^/ Pulmicort^®^ ((**a**) 210 nm for ipratropium bromide, (**b**) 246 nm for budesonide)), and (**D**) Atrovent^®^/ Fluimucil^®^ ((**a**) 210 nm for ipratropium bromide, (**b**) 214 nm for NAC)).

The contents of active components in test samples remained basically stable within the accepted range of HPLC error compared with those at the initial time after mixing (T_0_), with the exception that the content of NAC in Atrovent^®^/ Pulmicort^®^ showed a slight decrease after 0.5 h (Figure 2). The representative chromatograms of admixtures and single-drug preparations are presented in Figure 3 and Figure 4. It needs to be explained that the drifting baselines in some of the chromatograms were occasioned by the steep gradient elution. Taking Figure 4C as an example, the gradient elution of separating ipratropium bromide and budesonide (see Table 2) resulted in the drifting baseline at a short wavelength (Figure 4Ca, 210 nm) but no substantial change at the longer wavelength (Figure 4Cb, 246 nm). However, the drift at 210 nm did not interfere with the determination of ipratropium bromide. Similarly, the drifts caused by gradient elution could also be observed in Figure 3Fa and Figure 4Db. Except that the double peaks were assigned to the two isomers of budesonide at about 23 min in Figure 3Fa,c and Figure 4Ca, the rest of the unidentified peaks were solvent peaks.

### 3.2. Geometrical Particle Size Distribution

Table 3 shows that the X10 values of all test samples, including the single dose of each drug and the mixed inhalation solutions, were less than 2 μm; the X50 and VMD values were in the scope of 3 to 5 μm, and the X90 values ranged from 6 to 9 μm.

### 3.3. Aerosol Particle Size Distribution

**Figure 5:**
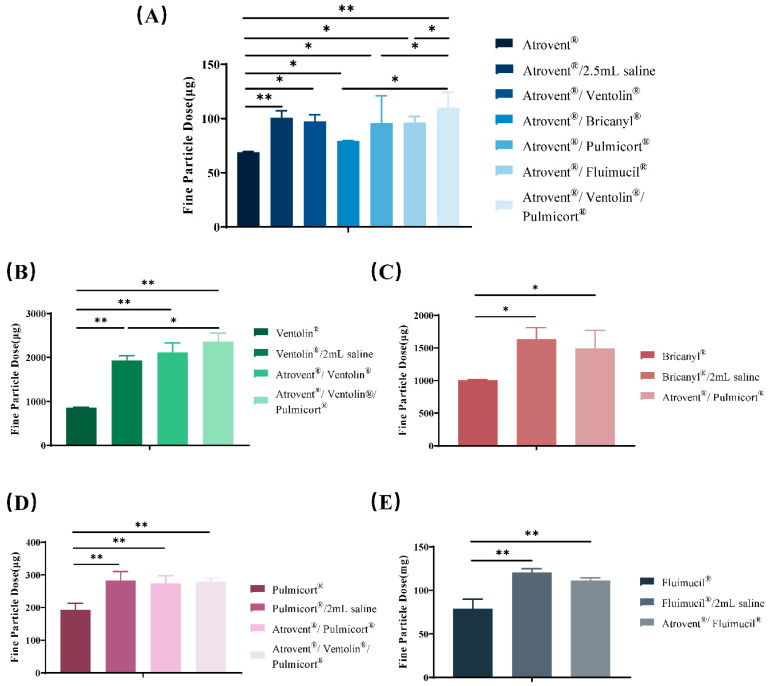
The FPD of ipratropium bromide (**A**), salbutamol sulfate (**B**), terbutaline sulfate (**C**), budesonide (**D**), and NAC (**E**) in admixtures or single-drug preparations. Results are shown as mean ± standard deviation (*n* = 5). Statistical calculations were performed using ANOVA multiple comparison. * and ** indicate *p* < 0.05 and *p* < 0.01.

The FPD values of each active component in the mixed inhalation solutions were consistent with the values of the saline control groups but showed a statistically significant increase (*p* < 0.05) compared with the single-drug preparations (Table 4 and Figure 5). On the other hand, the FPF and MMAD of admixtures remained unaffected in comparison with the two control groups, with the exception that the MMAD of budesonide exhibited a slight increase after mixing.

### 3.4. Active Drug Delivery Rate and Total Active Drug Delivered

**Figure 6:**
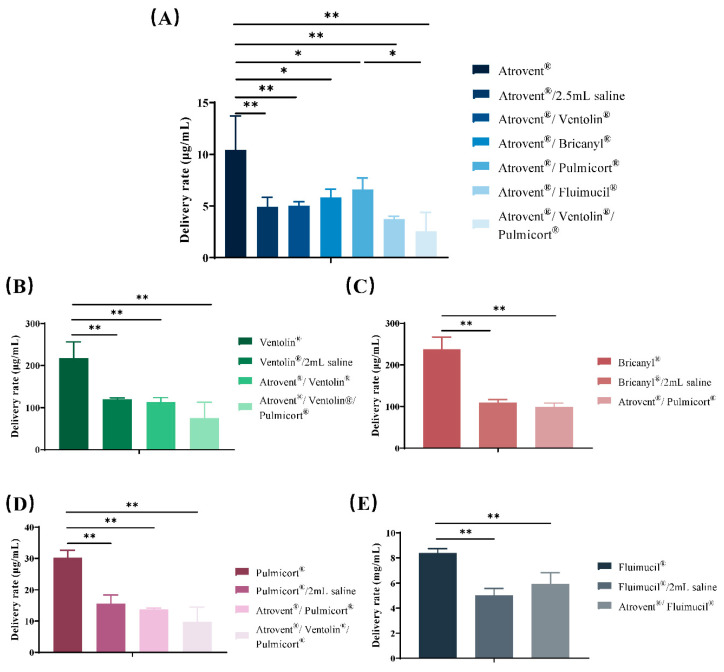
Active drug delivery rate of ipratropium bromide (**A**), salbutamol sulfate (**B**), terbutaline sulfate (**C**), budesonide (**D**), and NAC (**E**) in admixtures or single-drug preparations. Results are shown as mean ± standard deviation (*n* = 5) analyzed via ANOVA. * and ** indicate *p* < 0.05 and *p* < 0.01.

**Figure 7:**
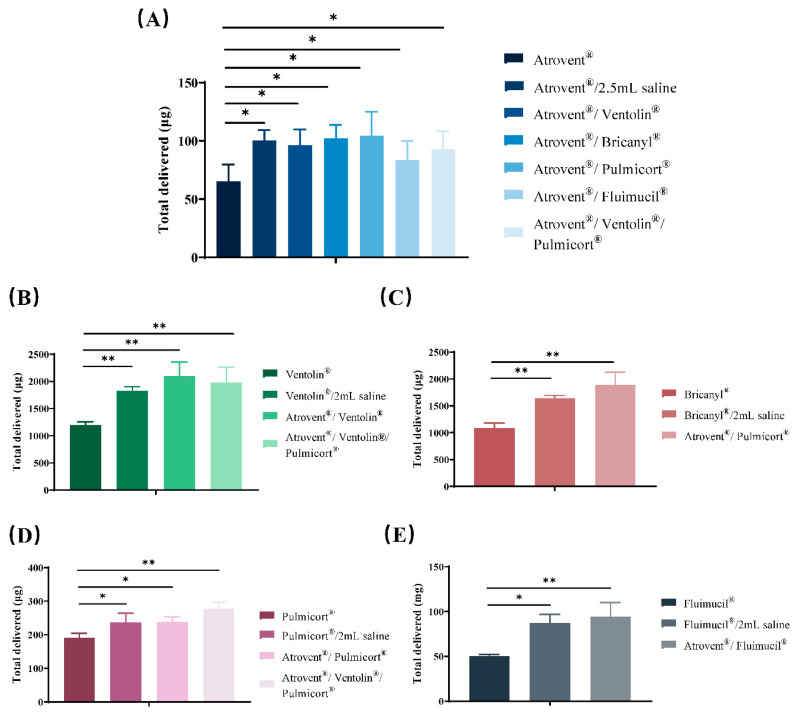
Total active drug delivered of ipratropium bromide (**A**), salbutamol sulfate (**B**), terbutaline sulfate (**C**), budesonide (**D**), and NAC (**E**) in admixtures or single-drug preparations. Results are shown as mean ± standard deviation (*n* = 5). * and ** indicate *p* < 0.05 and *p* < 0.01 by ANOVA.

Based on the data presented in Table 5 and Figure 6 and Figure 7, a significantly lower delivery rate and a higher amount of total active drug delivered were observed in admixtures compared with single-drug preparations. Additionally, no significant difference was found between admixtures and saline control groups in drug delivery characteristics.

## 4. Discussion

The administration of combined inhalation solutions is frequently used in the treatment of pulmonary diseases. At present, only a few publications have focused on the impact generated by simultaneous inhalation [10,12,14,29]. To our knowledge, this study is the first systematic one addressing the physicochemical compatibility and aerosol characteristics of Atrovent^®^ mixed with Ventolin^®^, Bricanyl^®^, Pulmicort^®^, and Fluimucil^®^.

In our study, variations in pH and osmolality were regarded as evaluation indicators of physical compatibility. The pH values of admixtures, which fell into the suitable range for inhalation, were laid between those of single-drug preparations. There was little meaningful change in the pH value of mixed inhalation solution within the short period of 1 h. Although the pH values of admixtures were stable during the testing time, the results might be quite different for longer timescales. Several studies suggest that, due to the susceptibility of the ester linkage in ipratropium bromide to hydrolysis, a high-pH environment (pH > 4) promotes the progress of hydrolysis of ipratropium bromide to tropic acid and a tropine derivative, leading to a more than 10% loss of ipratropium after 2 h [30,31]. Given this situation and the existing data, the mixed inhalation solutions, especially Atrovent^®^/Pulmicort^®^, Atrovent^®^/Fluimucil^®^, and Atrovent^®^/Ventolin^®^/Pulmicort^®^, should be used as soon as possible after mixing. Likewise, considering the slight decrease in NAC content, Atrovent^®^/Fluimucil^®^ should be used immediately to avoid efficacy reduction. Similar trends to those of pH values were seen regarding osmotic pressure. Mixing with Atrovent^®^ decreased the osmolarity of Fluimucil^®^ to a more appropriate level, reducing the occurrence of tissue irritation and coughing related to inhalation of hyperosmotic solutions [29]. Together with the experimental data on chemical compatibility, our work indicates that Atrovent^®^ is physiochemically stable with Ventolin^®^, Bricanyl^®^, Pulmicort^®^, and Fluimucil^®^.

While physicochemical properties remain stable, simultaneous inhalation may alter the aerosol characteristics including particle size distribution, active drug delivery rate, and total active drug delivered. No major change was observed in the geometrical particle size distribution determined by the laser diffraction method. A slight decrease in X90 was observed in all mixtures in comparison with single-drug preparations. The FPD values of mixed inhalation solutions escalated markedly (*p* < 0.05) compared to single-drug preparations. The obvious changes occurred because the total mass output by nebulization increased with the rinsing volume of inhalation solutions on account of the constant dead volume of the nebulization cup. This also corroborates the significant increase in total active drug delivered and the roughly unchanged FPF after mixing [13,32,33]. The lower concentration resulting from mixing and the limited nebulization volume in a fixed time were the two factors that were attributed to the substantial decrease in active drug delivery rate of admixtures compared to single-drug preparations. Besides the above, the similar results between admixtures and the saline control groups confirmed that it was the solution volume, rather than the drug by itself or excipients in formulations, that influenced the aerosol characteristics.

The present study has certain limitations. Firstly, an additional set of experiments should be executed to validate the results and further strengthen our conclusions. For instance, groups controlling excipients at working concentrations should be added to eliminate the effects of excipients. Meanwhile, the determinant role of solution volume in changing aerosol behavior could be confirmed by groups that keep solution volume constant for all mixture compositions. If the results of this experiment show that there is no significant difference in aerosol characteristics between relevant groups, this will support our conjecture. Secondly, since we believe that the dead volume of the nebulization cup has a great impact; different models or brands of nebulization devices with different dead volumes or different properties may produce different results in terms of aerosol parameters. Several studies have demonstrated that different nebulizer cups affect the character of the produced aerosol significantly, not only because of the dead volume but also because of the design and construction [34,35,36].

In conclusion, Atrovent^®^ is physically and chemically compatible with Ventolin^®^, Bricanyl^®^, Pulmicort^®^, and Fluimucil^®^ and simultaneous inhalation is more efficient since the FPD and total active drug delivered increase.

## Figures and Tables

**Table 1:** Volume and nebulization times of all samples.

Samples	Volume (mL)	Nebulization Time (min)
Atrovent^®^	2	8
Ventolin^®^	2.5	9
Bricanyl^®^	2	8
Pulmicort^®^	2	8
Fluimucil^®^	3	10
Atrovent^®^/Ventolin^®^	4.5	13
Atrovent^®^/Bricanyl^®^	4	13
Atrovent^®^/Pulmicort^®^	4	14
Atrovent^®^/Fluimucil^®^	5	14
Atrovent^®^/Ventolin^®^/Pulmicort^®^	6.5	19

**Table 2:** Chromatographic parameters.

Admixtures	Active Components	Ratio of Mobile Phase (A/B) by Volume	Injection Volume (μL)	Run Time (min)	Detection Wavelength (nm)
Atrovent^®^	ipratropium bromide	75/25	80	7.5	210
Ventolin^®^	salbutamol sulfate	75/25	20	4.5	276
Bricanyl^®^	terbutaline sulfate	75/25	20	4.5	276
Pulmicort^®^	budesonide	62/38	20	22	246
Fluimucil^®^	NAC	91/9	5	5	214
Atrovent^®^/Ventolin^®^	ipratropium bromide	75/25	80	7.5	210
	salbutamol sulfate				276
Atrovent^®^/Bricanyl^®^	ipratropium bromide	75/25	80	7.5	210
	terbutaline sulfate				276
Atrovent^®^/Pulmicort^®^	ipratropium bromide	0 min 75/25 8 min 75/25 12 min 55/45 16.5 min 55/45 19.5 min 65/35	80	25	210
	budesonide				246
Atrovent^®^/Fluimucil^®^	ipratropium bromide	75/25	80	7.5	210
	NAC	0 min 91/9 5 min 91/9 12 min 40/60	5	14.5	214
Atrovent^®^/Ventolin^®^/Pulmicort^®^	ipratropium bromide	0 min 75/25 8 min 75/25 12 min 55/45 16.5 min 55/45 19.5 min 65/35	80	25	210
	salbutamol sulfate				276
	budesonide				246

Abbreviation: NAC, *N*-acetylcysteine.

**Table 3:** X10, X50, X90, and VMD of test samples.

Samples	X10 (μm)	X50 (μm)	X90 (μm)	VMD (μm)
Atrovent^®^	1.67 ± 0.20	4.19 ± 0.08	7.01 ± 0.17	4.26 ± 0.14
Ventolin^®^	1.68 ± 0.11	4.32 ± 0.13	6.75 ± 0.16	4.24 ± 0.05
Bricanyl^®^	1.66 ± 0.06	4.40 ± 0.05	7.64 ± 0.18	4.55 ± 0.08
Pulmicort^®^	1.87 ± 0.09	4.37 ± 0.02	7.19 ± 0.17	4.97 ± 0.07
Fluimucil^®^	1.54 ± 0.13	4.03 ± 0.09	6.69 ± 0.11	4.18 ± 0.11
Atrovent^®^/Ventolin^®^	1.69 ± 0.10	3.93 ± 0.04	6.25 ± 0.16	3.95 ± 0.16
Atrovent^®^/Bricanyl^®^	1.68 ± 0.11	4.10 ± 0.12	6.82 ± 0.09	4.20 ± 0.04
Atrovent^®^/Pulmicort^®^	1.73 ± 0.07	4.02 ± 0.18	6.38 ± 0.04	4.03 ± 0.00
Atrovent^®^/Fluimucil^®^	1.55 ± 0.15	3.95 ± 0.08	6.89 ± 0.17	4.10 ± 0.13
Atrovent^®^/Ventolin^®^/Pulmicort^®^	1.70 ± 0.11	4.09 ± 0.13	6.61 ± 0.09	4.13 ± 0.12

Results are shown as mean ± standard deviation (*n* = 5). Abbreviation: VMD, volume median droplet diameter.

**Table 4:** FPD, FPF, and MMAD of test samples.

Samples	Active Components	FPD ^1^	FPF (%)	MMAD (μm)
Atrovent^®^	Ipratropium bromide	68.98 ± 0.28	66.40 ± 1.01	3.07 ± 0.11
Ventolin^®^	salbutamol sulfate	856.07 ± 16.23	59.90 ± 1.40	4.12 ± 0.13
Bricanyl^®^	terbutaline sulfate	1004.28 ± 9.35	58.33 ± 2.27	3.82 ± 0.20
Pulmicort^®^	budesonide	193.14 ± 19.80	47.65 ± 1.77	5.06 ± 0.16
Fluimucil^®^	NAC	78.91 ± 11.04	55.38 ± 7.21	4.44 ± 0.58
Atrovent^®^/2.5 mL saline	Ipratropium bromide	100.72 ± 6.37	63.92 ± 2.49	3.39 ± 0.03
Ventolin^®^/2 mL saline	salbutamol sulfate	1934.29 ± 103.40	63.02 ± 1.25	3.42 ± 0.18
Bricanyl^®^/2 mL saline	terbutaline sulfate	1630.02 ± 180.39	60.32 ± 1.96	3.28 ± 0.28
Pulmicort^®^/2 mL saline	budesonide	282.29 ± 27.93	46.20 ± 3.05	5.30 ± 0.84
Fluimucil^®^/2 mL saline	NAC	120.83 ± 4.07	52.40 ± 2.04	4.38 ± 0.06
Atrovent^®^/Ventolin^®^	ipratropium bromide	97.23 ± 6.37	65.54 ± 1.80	3.15 ± 0.06
	salbutamol sulfate	2112.95 ± 217.01	65.27 ± 0.98	3.18 ± 0.07
Atrovent^®^/Bricanyl^®^	ipratropium bromide	99.28 ± 0.17	64.71 ± 0.04	3.63 ± 0.02
	terbutaline sulfate	1491.85 ± 276.90	61.79 ± 2.80	3.46 ± 0.17
Atrovent^®^/Pulmicort^®^	ipratropium bromide	95.90 ± 25.08	50.50 ± 8.94	3.89 ± 0.77
	budesonide	274.23 ± 23.13	47.01 ± 4.91	6.45 ± 0.49
Atrovent^®^/Fluimucil^®^	ipratropium bromide	96.17 ± 5.81	54.73 ± 1.30	4.48 ± 0.15
	NAC	111.61 ± 2.86	53.39 ± 1.64	4.55 ± 0.17
Atrovent^®^/Ventolin^®^/Pulmicort^®^	ipratropium bromide	120.16 ± 4.18	57.97 ± 5.32	4.68 ± 0.43
	salbutamol sulfate	2359.09 ± 198.30	52.66 ± 4.23	4.68 ± 0.33
	budesonide	279.87 ± 10.79	44.78 ± 1.76	6.29 ± 0.27

^1^ Concentrations of FPD are mg for Fluimucil^®^, Fluimucil^®^/2 mL saline, and Atrovent^®^/Fluimucil^®^ and μg for all other groups. Results are shown as mean ± standard deviation (*n* = 5). Abbreviations: FPD, fine particle dose; FPF, fine particle fraction; MMAD, mass medium aerodynamic diameter.

**Table 5:** Active drug delivery rate and total active drug delivered of test samples.

Samples	Active Components	Active Drug Delivery Rate ^1^	Total Active Drug Delivered ^2^
Atrovent^®^	Ipratropium bromide	10.43 ± 3.30	65.34 ± 14.37
Ventolin^®^	salbutamol sulfate	218 ± 38.50	1201.00 ± 56.20
Bricanyl^®^	terbutaline sulfate	237.96 ± 29.20	1084.73 ± 95.26
Pulmicort^®^	budesonide	30.27 ± 2.33	190.46 ± 14.13
Fluimucil^®^	NAC	8.39 ± 0.35	50.14 ± 1.91
Atrovent^®^/2.5 mL saline	Ipratropium bromide	4.93 ± 0.92	100.39 ± 8.92
Ventolin^®^/2 mL saline	salbutamol sulfate	120.37 ± 3.24	1830.47 ± 72.39
Bricanyl^®^/2 mL saline	terbutaline sulfate	109.49 ± 7.34	1640.28 ± 50.76
Pulmicort^®^/2 mL saline	budesonide	15.57 ± 2.78	237.33 ± 27.07
Fluimucil^®^/2 mL saline	NAC	5.04 ± 0.53	86.99 ± 9.67
Atrovent^®^/Ventolin^®^	ipratropium bromide	5.05 ± 0.36	96.32 ± 13.57
	salbutamol sulfate	113.53 ± 10.27	1936 ± 53.87
Atrovent^®^/Bricanyl^®^	ipratropium bromide	5.83 ± 0.80	102.22 ± 11.48
	terbutaline sulfate	99.41 ± 9.29	1694.74 ± 29.66
Atrovent^®^/Pulmicort^®^	ipratropium bromide	6.58 ± 1.14	104.27 ± 20.94
	budesonide	13.74 ± 0.44	237.87 ± 35.50
Atrovent^®^/Fluimucil^®^	ipratropium bromide	3.73 ± 0.29	83.39 ± 16.60
	NAC	5.93 ± 0.91	94.02 ± 16.09
Atrovent^®^/Ventolin^®^/Pulmicort^®^	ipratropium bromide	2.55 ± 1.84	92.90 ± 15.32
	salbutamol sulfate	75.01 ± 37.75	1981.51 ± 283.01
	budesonide	9.76 ± 4.77	277.82 ± 19.42

^1^ Concentrations are mg/min for Fluimucil^®^, Fluimucil^®^/2 mL saline, and Atrovent^®^/ Fluimucil^®^ and μg/min for all other groups. ^2^ Concentrations are mg for Fluimucil^®^, Fluimucil^®^/2 mL saline, and Atrovent^®^/ Fluimucil^®^ and μg for all other groups. Results are shown as mean ± standard deviation (*n* = 5).
